# Case presentation: implantation of cardiac resynchronization therapy pacemaker via the coronary sinus in a patient with triple valve replacement

**DOI:** 10.1186/s12872-018-0775-7

**Published:** 2018-02-21

**Authors:** Cheng Zheng, Wei-Qian Lin, Yuan-Zheng Lin, Hao Lian, Zhi-Rui Liu, Jia-Hui Chen, Jia-Feng Lin

**Affiliations:** 1 Department of Cardiology, Second Affiliated Hospital and Yuying Children’s Hospital of Wenzhou Medical University, 109 Xueyuan Road, Wenzhou, Zhejiang 325000 China; 2 Department of Cardiology, Taishun general hospital, Wenzhou, 325000 China

**Keywords:** Cardiac resynchronization therapy pacemaker, Triple valve replacement, Narrow QRS duration, Ventricular synchrony

## Abstract

**Background:**

In patients with triple valve replacement developing third-degree atrioventricular block (AVB), the most appropriate approach for permanent pacemaker implantation remains questionable.

**Case presentation:**

In this case presentation, we first described the approach of implantation of the cardiac resynchronization therapy pacemaker (CRT-P) via one bipolar pacing lead in middle cardiac vein (MCV) and one quadripolar pacing lead in anterior interventricular vein (AIV) in a patient developing complete AVB, who had been previously diagnosed with rheumatic valvular heart disease with triple valve replaced. After the CRT-P implantation, the two pacing leads in coronary sinus (CS) provided a dual-site ventricular pacing from the anterior septum and posterior septum, which resulted in a narrow QRS complex and an increased ventricular synchrony. During the long-term follow-up, no deterioration of heart function was documented and pacing parameters remained good.

**Conclusion:**

In this patient developing complete AVB with triple valve replaced, our approach of CRT-P implantation provides an effective and reliable ventricular pacing, and is an alternative option when transvenous right ventricular pacing, transseptal left ventricular pacing and transpericardial epicardium pacing are not possible. Further prospective randomized trials are required to confirm the efficiency of our approach of dual-site ventricular pacing by CRT-P in this kind patients.

## Background

In rheumatic heart disease patients with triple mechanical valve replacement and complete atrioventricular block (AVB), the most appropriate and beneficial method of pacemaker implantation remains questionable. With the tricuspid mechanical valve replaced, endocardial pacing of the right ventricle is not possible because such a manipulation may cause disastrous damage to the prosthetic valve [1]. In addition, left ventricular endocardial pacing via the atrial septum puncture was contraindicated due to the replacement of the mitral mechanical valve [2]. Epicardial pacing was also limited because of rheumatic pericardial adhesion and unsatisfying pacing parameters [3]. In this kind patients, a single lead in the coronary sinus (CS) to pace the left ventricle has been reported [3–5], however, it may lead ventricular dyssynchronization, and in the event of CS lead dislodgement or malfunction, the underlying complete AVB could be life-threatening. Here, we presented a case where we applied dual-site ventricular pacing by cardiac resynchronization therapy pacemaker (CRT-P) implantation, with one bipolar pacing lead in the middle cardiac vein (MCV) and one quadripolar pacing lead in the anterior interventricular vein (AIV) to simultaneously stimulate the ventricle in a patient with triple valve replacement.

## Case presentation

A 42-year-old woman with a history of rheumatic valvular heart disease and permanent atrial fibrillation underwent open triple valvotomy 14 years ago and routinely took the warfarin to anti-coagulate since then. The international normalized ratio of prothrombin time (PT-INR) was monitored every 2 weeks to keep it range between 2.5 and 3.5 s. She complained of persistent abdominal distension and repeated exertional dyspnea for 2 years at admission. Physical examination showed obvious body signs of right heart failure, including jugular vein distention, lower limb pitting edema. The 12-lead surface ECG showed atrial fibrillation rhythm with narrow QRS complex. The transthoracic echocardiography indicated a normal ventricular size of 35 mm with left ventricular ejection fraction (LVEF) of 65% and a moderate stenosis of tricuspid valve with the pressure gradient across the tricuspid valve of 8 mmHg. Laboratory examination revealed the NT-pro-BNP of 1780 ng/ml. Due to progression of heart failure from her valvular heart disease, she underwent tricuspid valve replacement again (Medtronic bileaflet prosthesis). On the second day after surgery, the patient developed sudden cardiac arrest with bedside electrocardiographic monitor recording atrial fibrillation, complete third-degree AVB with repeated episodes of torsade de point secondary to the long QT interval (0.6 s). Thus, a permanent pacemaker was required to oppose this malignant arrhythmia.

**Fig. 1:**
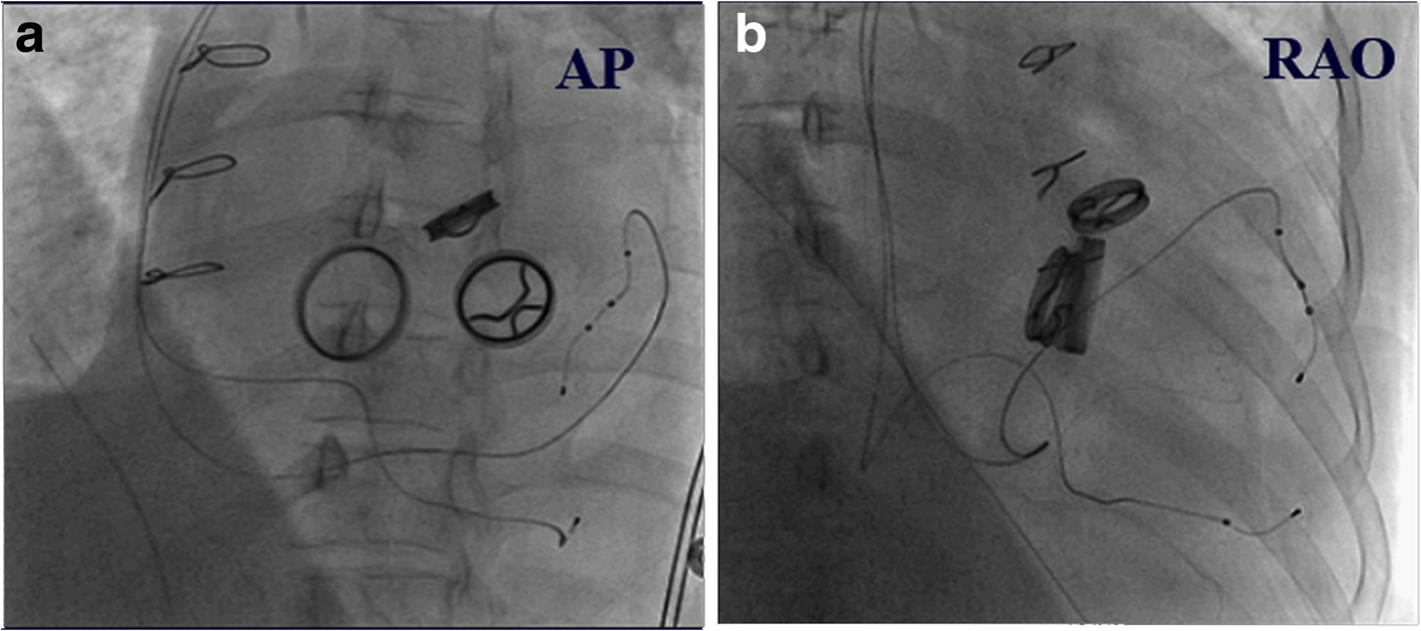
A quadripolar pacing lead (Quartet LV lead model 1458Q, StJude Medical) was introduced into anterior interventricular vein (AIV), while a bipolar pacing lead (QuickFlex μ model 1258 T, St Jude Medical) into middle cardiac vein (MCV). **a**. Anteroposterior position. **b**. Right anterior oblique position

**Fig. 2:**
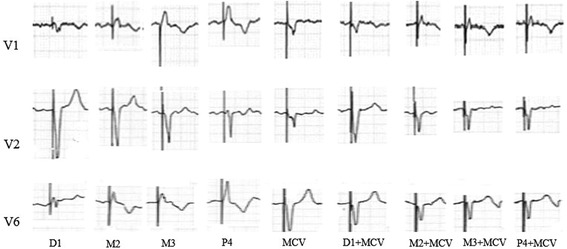
The procedure of pacing interval optimization between the two pace leads

**Fig. 3:**
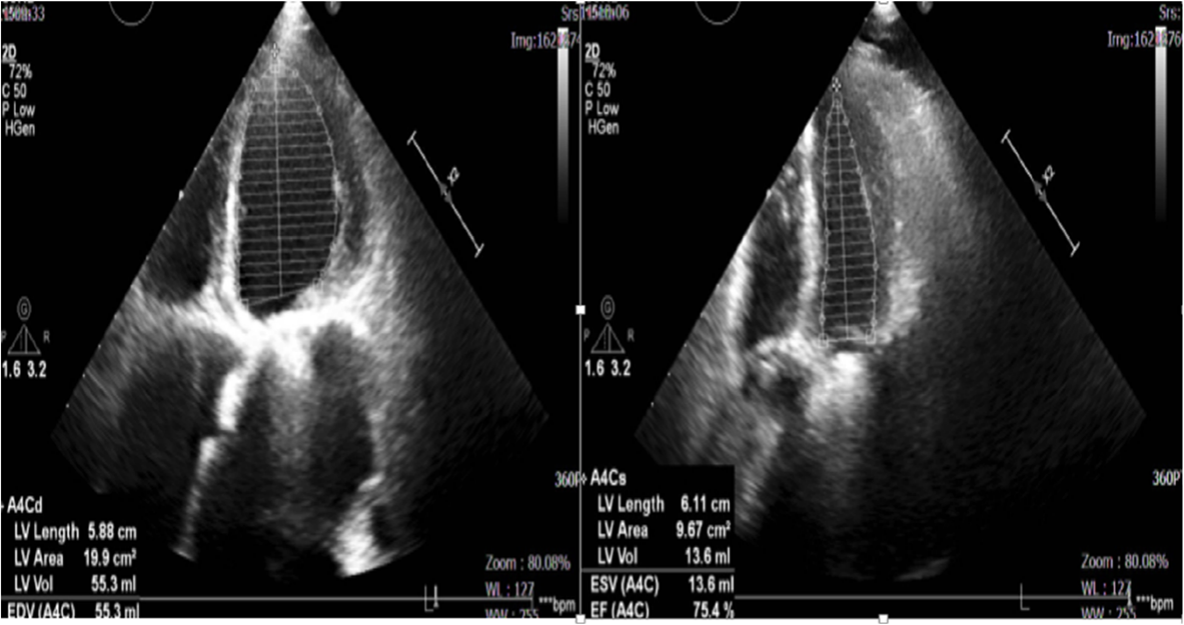
Systolic heart function evaluated by Simpsons method

After taking full consideration of the patient’s basic situations, the CRT-P implantation via two pacing leads in cardiac venous system to provide dual-site ventricular pacing was primarily planned. As the patient with triple mechanical valve replaced had a relatively higher risk for thrombosis, the oral warfarin was not interrupted before operation. The instant blood test prior to operation showed a PT-INR of 2.42 s. The intravenous unfractionated heparin was used during the procedure to keep an activated clotting time (ACT) range between 250 and 300 s. Electric surgical knives were applied to stop bleeding of the incision during the whole procedure. A guiding catheter was delivered via left subclavian vein puncture to access the coronary sinus ostium, which was located between the inferior vena cava and the mechanical tricuspid valve on the inferior aspect of the atrial septum. A retrograde occlusion venogram was then performed to evaluate the anatomy of the cardiac venous system. As the lateral cardiac vein was too thin and the posterolateral vein was too tortuous for pacing leads to access, the MCV and the AIV were selected as target vessels for pacing leads placement. A quadripolar pacing lead (Quartet LV lead model 1458Q, St Jude Medical) was introduced into the AIV, while bipolar pacing lead (Quick Flex μ model 1258 T, St Jude Medical) into the MCV, both leads were secured to the pectoral muscle fascia and attached to a CRT-P (Allure Quadra™ CRT-P, St Jude Medical), Fig. 1. For the patient was in the permanent atrial fibrillation rhythm, the atrial lead placement was abandoned due to the inability to achieve an atrioventricular synchrony. The pacing rate was initially set at 100 bpm in a VVI pacing mode (ventricle paced, ventricle sensed, pacing is inhibited if beat is sensed). By the procedure of pacing interval optimization between the two pacing leads, we obtained a relatively narrowest QRS duration of 116 ms among all bipolar and unipolar pacing configurations, using the vector from the proximal tip (P4) of quadripolar lead to the coil of bipolar lead in MCV, vector P4–MCV coil, Fig. 2 and Table 1. At the beginning of the pacemaker implantation, the quadripolar lead paced with a capture threshold of 1.75 V at 1.0 ms pulse width with an impedance of 430 Ω, while the bipolar lead paced with a capture threshold of 1.5 V at 1.0 ms pulse width with an impedance of 600 Ω, using the vector P4–MCV coil. After the operation, the compression of pacemaker pocket with elastic bandage was immediately performed and removed after 48 h, no pocket hematoma and other bleedings was found, the PT-INR was measured of 2.28 s 24 h later. The left ventricular systolic function was evaluated with Simpsons method by transthoracic echocardiography within the first 24 h after pacemaker implantation. The ventricular dual-site pacing exhibited an obvious acute hemodynamic improvement over single-site pacing (LVEF: ventricular dual-site pacing 71.5 ± 0.96% vs ventricular single-site pacing 62.9 ± 2.38%), and the best left ventricular systolic function was obtained by ventricular dual-site pacing of P4 combined with MCV, Table 2. A remarkable decline in pacing threshold of both leads was found 2 months later, the quadripolar lead paced with a capture threshold of 0.75 V at 0.5 ms pulse width with an impedance of 490 Ω while the bipolar lead paced with a capture threshold of 1.25 V at 0.8 ms pulse width with an impedance of 550 Ω using the vector P4–MCV coil. During one-year follow-up, the patient was VVI pacing mode dependent with an intrinsic heart rhythm of atrial fibrillation with complete AVB. She did not present deteriorations of heart function in transthoracic echocardiography (LVEDD 40 mm, LVEF 75.4%, see Fig. 3) with 6-min walking test recording a walking distance more than 450 m, meanwhile, CRT programming control showed good pacing parameters all the time.

**Table 1:** QRS duration produced by pacing interval optimization

Vector	D1	M2	M3	P4	MCV	D1 + MCV	M2 + MCV	M3 + MCV	P4 + MCV	Basic
QRS (ms)	128	126	124	123	134	126	122	120	116	120

**Table 2:** Left ventricular systolic ejection fraction produced by different pacing sites

Pacing site	D1	M2	M3	P4	MCV	D1 + MCV	M2 + MCV	M3 + MCV	P4 + MCV	basic
Ejection (%)	64.3	58.8	64.4	64.2	62.9	72.2	70.4	71.0	72.4	55

## Discussion

In this case, we first described the method of implantation of CRT-P with two pacing leads in coronary venous system, which provided an effective dual-site ventricular pacing in a patient developing complete atrioventricular block after triple mechanical valves replacement.

In this patient, as the mechanical tricuspid and mitral valves implanted, placing pacing lead in right ventricular endocardium across the tricuspid valve was forbidden because of underlying risk of valve damage and failure, in addition, delivering pacing lead to left ventricular endocardium via the atrial septum puncture was also contraindicated due to the potential damage to the mitral valve [1, 2]. The method of placing an epicardial pacing lead via the thoracotomy was technically challenging, because of severe pericardial adhesion caused by the rheumatic heart disease and previous surgery in this patient, meanwhile, in such a situation, long-term epicardial pacing tended to be unreliable with high pacing thresholds [3]. The method of the coronary sinus single-site ventricular pacing was also taken into consideration [4]. Recently, a retrospective clinical study reported that in patients with tricuspid valvular replacement, coronary sinus single-site left ventricular pacing was a feasible alternative to right apical ventricular pacing. However, this study also revealed that long-term coronary sinus single-site pacing resulted in an inferior left ventricular systolic function compared with standard cardiac resynchronization therapy, in addition, in their study, a patient of long-term coronary sinus single-site ventricular pacing was found developing pacing-induced cardiomyopathy with progressive decline in LVEF [5]. Moreover, in this patient of complete AVB, if single pacing lead in coronary sinus was selected, in the event of coronary sinus lead dislodgement or malfunction, the underlying complete AVB could be life-threatening. Based on these considerations, the idea of CS single-site pacing was abandoned. The outcomes of BLOCK-HF research indicated that the superior long-term clinical and functional benefit of dual-site ventricular pacing in patients with AV block and mild/moderate heart dysfunction compared with single-site ventricular pacing [6]. Thus, in this patient, after full consideration, the dual-site ventricular pacing via coronary sinus method was primarily chosen. Different from classical cardiac resynchronization therapy with dual-site pacing (LV free wall pacing from the coronary venous system in conjunction with RV apical pacing), in this patient, we performed dual-site ventricular pacing via coronary sinus by implantation of CRT-P with one bipolar lead in the MCV and one quadripolar lead in the AIV, which provided an anterior ventricular wall pacing in conjunction with a posterior ventricular wall pacing. By our method of ventricular dual-site pacing in coronary sinus, increased ventricular electrical and mechanical synchronization was obtained, long-term follow up also showed a good clinical outcome. However, before our approach of dual-site CS pacing can be recommended to more patients, a prospective study is needed to prove this preliminary finding.

In this patient, due to a relatively higher thrombotic risk associated with triple mechanical valve replacement, the oral anticoagulant therapy of warfarin was not interrupted and a bridging therapy with low-molecular-weight heparin (LMWH) was not initiated. Though this protocol was some different from the standard anticoagulation protocol at the time of pacemaker implantation, which required stopping oral anticoagulation therapy and initiating a bridging therapy with subcutaneous LMWH, no pocket hematoma and thromboembolic events was found in this patient. Our observation further confirmed the safety of anticoagulation protocol proposed by the POCKET study [7].

## Conclusions

CRT-P implantation with two pacing leads in coronary sinus can provide an effective and reliable dual-site ventricular pacing. Further prospective randomized trials are required to confirm the efficiency of our approach of dual-site ventricular pacing by CRT-P.

## Abbreviations

ACT: Activated clotting time
AIV: Anterior interventricular vein
AVB: Atrioventricular block
CRT-P: Cardiac resynchronization therapy pacemaker
CS: Coronary sinus
LMWH: Low-molecular-weight heparin
LVEDD: Left ventricular end diastolic diameter
LVEF: Left ventricular ejection fraction
MCV: Middle cardiac vein
PT-INR: International normalized ratio of prothrombin time
VVI: ventricle paced, ventricle sensed, pacing is inhibited if beat is sensed
